# Synergistic Flame Retardant Effect between Ionic Liquid Functionalized Imogolite Nanotubes and Ammonium Polyphosphate in Epoxy Resin

**DOI:** 10.3390/polym15061455

**Published:** 2023-03-15

**Authors:** Taohua Zhu, Xuan Zhou, Guozheng Guo, Zhihua Chai, Ming Gao

**Affiliations:** 1 Department of Chemistry and Chemical Engineering, School of Chemistry and Biological Engineering, University of Science and Technology Beijing, Beijing 100083, China; 2 School of Electronic Science and Control Engineering, Institute of Disaster Prevention, Sanhe 065201, China; 3 Heibei Key Laboratory of Hazardous Chemicals Safety and Control Technology, School of Chemical Safety, North China Institute of Science and Technology, Sanhe 065201, China

**Keywords:** INTs-PF_6_-ILs, synergistic effect, epoxy resin, flame retardancy

## Abstract

Ionic liquid functionalized imogolite nanotubes (INTs-PF_6_-ILs) were introduced into the epoxy resin (EP)/ammonium polyphosphate (APP) system to investigate the flame retardant performance and thermal properties using the limiting oxygen index (LOI) test, the UL-94 test, and the cone calorimeter test (CCT). The results suggested that a synergistic effect exists between INTs-PF_6_-ILs and APP on the char formation and anti-dripping behavior of EP composites. For the EP/APP, an UL-94 V-1 rating was obtained for the loading of 4 wt% APP. However, the composites containing 3.7 wt% APP and 0.3 wt% INTs-PF_6_-ILs could pass the UL-94 V-0 rating without dripping behavior. In addition, compared with the EP/APP composite, the fire performance index (FPI) value and fire spread index (FSI) value of EP/APP/INTs-PF_6_-ILs composites were remarkably reduced by 11.4% and 21.1%, respectively. Moreover, the char formed by EP/APP composites was intumescent, but of poor quality. In contrast, the char for EP/APP/INTs-PF_6_-ILs was strong and compact. Therefore, it can resist the erosion due to heat and gas formation and protect the inside of the matrix. This was the main reason for the good flame retardant property of EP/APP/INTs-PF_6_-ILs composites.

## 1. Introduction

Epoxy resin (EP), with its excellent mechanical properties, chemical resistance, electrical insulation, adhesion, and low manufacturing cost, has been recognized as a material of high value in various fields, such as coatings, transportation, electronic appliances, and high performance composite materials [1,2,3]. However, the limiting oxygen index (LOI) of EP is only about 20% and released a large amount of smoke during combustion [4,5]. The flammability of EP limits its application in fields, such as construction, coal mines, and other occasions with high requirements on flame retardancy [6]. Therefore, improving the flame retardancy of EP is significant. To date, halogen compounds are widely used in commercial settings due to its high flame retardancy efficiency, which can impart excellent flame-retardant properties to polymer materials. The disadvantage of halogen flame retardants is their potential health and environmental hazards, producing large amounts of corrosive and toxic substances during combustion [7,8]. Considering environmental pollution, halogen-free flame retardants, including phosphorus [9,10], nitrogen [11], silicon, and bio-based materials [12] and nanofillers [13,14,15], have been developed rapidly.

Ammonium polyphosphate (APP) is an important component of inorganic phosphorous flame-retardant. It is widely used in flame-retardant plastics, fibers, and rubber due to its advantages of low price, high contents of P and N, good thermal stability, durable flame-retardant performance, and low toxicity [16]. However, according to previous work, the flame retardant effect of the single use of APP in EP is not significant. It is necessary to use a suitable synergist in combination with APP to improve the fire resistance. In recent years, nano-fillers, such as carbon nanotubes, graphene, etc., have acquired special attention since a small weight percentage of this introduction can produce a nanocomposite with a notable improvement in flame retardance performance without affecting the mechanical properties, resulting in making these fillers attractive. Among the nano-fillers used for the preparation of EP nanocomposites, there may be imogolite nanotubes (INTs). INTs are a single-walled nanotube material consisting of hydrous aluminosilicate, which is composed of Al_2_-μOH in outer surface and Si-OH in inner surface [17,18]. Moreover, the stoichiometry of INTs is (OH)_3_Al_2_O_3_SiOH. Considering the element composition and tubular structure, which is similar to carbon nanotubes, INTs are possible as a potential synergistic flame retardant. However, few reports have been published regarding the synergistic effect of INTs and APP in the EP system.

In our recent work, INTs were prepared successfully and it was found that INTs were stacked together and could not be scattered. Then, INTs were modified with an ionic liquid of 1-butyl-3-methylimidazolium hexafluorophosphate ([BMIM]PF_6_) to improve its dispersion in hydrophobic polymer [19]. Moreover, the obtained product of INTs-PF_6_-ILs was well dispersed, spider-web-like, and composed of individual fibers according to the results of transmission electron microscope. Furthermore, the dispersion was indeed greatly improved after modification with [BMIM]PF_6_ [20]. In addition, as a synergistic flame retardant, INTs-PF_6_-ILs have achieved good flame retardancy with APP in unsaturated polyester resin (UPR). With the same loading of flame retardant, the UPR/APP/INTs-PF_6_-ILs composites can pass UL-94 V-0 rating and the LOI value was improved to 28.0. Moreover, the maximum mass loss rate, heat release rate, and total heat release were significantly reduced from the CCT results for the introduction of 0.4 wt% INTs-PF_6_-ILs [20].

Inspired by our recent work, INTs-PF_6_-ILs were introduced to EP with APP, in order to investigate the synergistic effect. Limiting oxygen index (LOI) instrument, UL-94 vertical burner, and cone calorimetry were used to study the flame retardant properties of the EP composites. Thermogravimetric analysis (TGA) was used to investigate the thermal degradation process for the EP composites. In addition, scanning electron microscopy (SEM) was employed to observe the micro-morphology of the char residue.

## 2. Experimental

### 2.1. Materials

Tetraethyl orthosilicate (TEOS, AR) and NaOH (AR) were purchased from Sinopharm Chemical Reagent Co., Ltd. (Shanghai, China). NH_3_·H_2_O (25%, aq) and Al(NO_3_)_3_·9H_2_O (AR) were purchased from Tianjin Yongda Chemical Reagent Co., Ltd. (Tianjin, China) and 1-butyl-3-methylimidazolium hexafluorophosphate ([BMIM]PF_6_, AR) was provided by Hubei Jiufeng Chemical Co., Ltd. (Wuhan, China). Anhydrous ethanol (AR) was provided by Jiangsu Qiangsheng Functional Chemical Co., Ltd. (Suzhou, China) and the silane coupling agent of (C_2_H_5_O)_3_-Si-(CH_2_)_3_NH_2_ (KH550, GR) was supplied by Shandong Huanzheng Chemical Co., Ltd. (Jinan, China). M-phenylenediamine (MPDA, AR) was obtained from Shanghai Aladdin Bio-Chem Technology Co., Ltd. (Shanghai, China) and EP at an industrial grade was provided by Henan Zhongyi Yiyuan Chemical Co., Ltd. (Zhengzhou, China). Finally, APP was obtained from the Tangshan Yongfa flame retardant material factory (Tangshan, China).

Moreover, INTs were prepared in the laboratory using the method of Arancibia-Miranda [21], and INTs-PF_6_-ILs were prepared in the laboratory using the method of Wan, M [22].

### 2.2. Preparation of EP Composites

EP was heated to 60 °C in a water bath to reduce its viscosity, and the flame retardant (APP or INTs-PF_6_-ILs) was slowly added to the epoxy monomer liquid with magnetic stirring for uniform. Curing agent MPDA was added, and then the mixture was stirred at high speed for 5 min. Finally, the mixture was poured into a mold to cure at 60 °C for 8 h. After it was cooled to room temperature, the samples were cut into standard sample bars for UL-94, CCT, and LOI tests. The samples of EP composites were presented in Table 1.

### 2.3. Measurement and Characterization of EP Composites

#### 2.3.1. Limiting Oxygen Index (LOI)

The LOI value was measured by the JF-3 oxygen index instrument (Jiangning Analysis Instrument Company, Nanjing, China) in accordance with ASTM D2863-97, and the specimen size was 100.0 × 6.5 × 3.0 mm. The results of LOI have been repeated for a total of three times.

#### 2.3.2. The Vertical Burning Test (UL-94)

The vertical burning test was determined by the CZF-3 instrument (Jiangning Analysis Instrument Co., Nanjing, China) in accordance with ASTM D3801, and the specimen size was 100.0 × 13.0 × 3.0 mm. The results of UL-94 have been repeated for a total of three times.

#### 2.3.3. Thermogravimetric Analysis (TGA)

TGA of EP composites was carried out using an HCT2 instrument (Shanghai Artisan Instrument Technology Co., Ltd., Shanghai, China) under air atmosphere over a temperature range from 50 to 735 °C with a heating rate of 15 °C/min. The mass of each sample was around 5 mg.

#### 2.3.4. Cone Calorimeter Test (CCT)

The cone calorimeter test (CCT) was performed using a PX-07-007 cone calorimeter (Phoenix quality inspection instrument Co., Ltd. (Suzhou, China) in accordance with the ISO 5660 standard under a heat flux of 50 kW/m^2^. The sheets for the test were 100.0 × 100.0 × 3.0 mm in dimension. Each specimen was wrapped in aluminum foil and exposed horizontally. This experiment was repeated for a total of three times and the results were similar to each other; therefore none of them was selected.

#### 2.3.5. Scanning Electron Microscopy (SEM)

The morphology of the residues after CCT was observed by the scanning electron microscope (SEM, KYKY-EM3200, Beijing Zhongke Keyi Co., Ltd. (Beijing, China)) with an accelerating voltage of 20 kV. Moreover, all the carbon layers were treated with gold spray before the observation.

## 3. Results and Discussion

### 3.1. LOI and UL-94 Rating of EP Composites

LOI and UL-94 tests were the most common methods to evaluate the flame retardancy of polymers. To examine the effectiveness of the flame retardant in the EP composites, the LOI and UL-94 tests were performed, and the results were shown in Table 2. From these results, it could be seen that the LOI value of the pure EP was very low, only 22.9, and combustion was accompanied by heavy dripping; therefore, it could not pass the UL-94 test. By introducing 4 wt% APP into EP, the LOI value of EP/APP composite was increased to 27.4, and the dripping behavior was modified. However, the UL-94 vertical combustion level was only V-1 due to the long flame time. In contrast, the presence of the INTs-PF_6_-ILs could dramatically increase the LOI value for EP. The introduction of only 0.3 wt% INTs-PF_6_-ILs and 3.7 wt% APP into EP generated a composition with an LOI value of 28.0, which increased by 0.6% compared with the EP/APP composite. In addition, the UL-94 rating for this material was V-0 and combustion was accompanied without dripping. Therefore, the flame retardancy of EP/APP/INTs-PF_6_-ILs composite had been clearly improved compared with the EP/APP composite, and the results proved that INTs-PF_6_-ILs had good synergistic flame retardancy with APP in EP.

### 3.2. Thermal Stability of EP Composites

**Figure 1:**
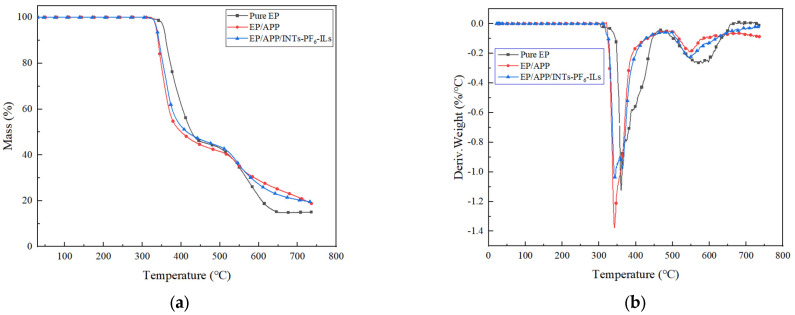
(**a**) TGA curves of EP composites; (**b**) DTG curves of EP composites.

TGA and differential thermogravimetry analysis (DTG) curves of the EP composites were shown in Figure 1a,b, respectively, and the related data were summarized in Table 3. The temperature at a 5 wt% weight loss was defined as the initial decomposition temperature (T_i_), and T_max_ was the temperature at the maximum weight loss rate.

As shown in Figure 1a, the decomposition of all EP composites had two steps. Pure EP lost weight rapidly from 280 to 430 °C in the first step since the oxygen-containing groups in pure EP decompose into CO, CO_2_, and H_2_O. The thermosetting property of the EP was decreased, and the multi-aromatic structure was formed in the carbon residue [23]. Weight loss of pure EP occurs from 430 to 650 °C in the second step, which was due to the oxidation decomposition of residual carbon with a multi-aromatic structure formed at high temperature in the air.

The T_i_ values of EP/APP and EP/APP/INTs-PF_6_-ILs composites were 336 and 337 °C, respectively, which was significantly lower than the pure EP at 355 °C. The T_max_ values of EP/APP and EP/APP/INTs-PF_6_-ILs composites were 342 and 343 °C, respectively, which was also significantly lower than the pure EP at 359 °C. These results were mainly due to the thermal degradation of APP at low temperature, resulting in the reduction in T_i_ in EP composites. APP was decomposed into polyphosphoric acid and ammonia gas during combustion [24,25], and polyphosphoric acid could react with hydroxyl or other oxygen-containing groups of EP to form unstable phosphate ester [26,27]. Moreover, with the increase in temperature, the phosphate ester would dehydrate to form char residue. As an effective flame retardant, the earlier it played a catalytic role in forming an expanded char layer, the more favorable it would be for the flame retardant [28].

The char residues of pure EP, EP/APP, and EP/INTs-PF_6_-ILs composites at 735 °C are 15.06, 18.83, and 19.61 wt%, respectively. Compared with the pure EP, the char residues of EP/APP and EP/INTs-PF_6_-ILs composites were increased by 3.77% and 4.55%, respectively, as the APP can promote the dehydration of EP to form char under high temperature. Compared with EP/APP, the char residues of EP/APP/INTs-PF_6_-ILs composites were increased by 0.78%. In addition, the T_i_ and T_max_ values of EP/APP/INTs-PF_6_-ILs composites were higher than the EP/APP composites, which could be clearly observed from Figure 1a. Between 300 and 540 °C, the line of EP/APP/INTs-PF_6_-ILs composites was always above the line of EP/APP composites. These results were observed since the addition of INTs-PF_6_-ILs has improved the thermal stability of the EP composite at low temperature. In contrast, above 540 °C, the line of EP/APP/INTs-PF_6_-ILs composites was below the line of EP/APP composites. Since at the time that the composite was burned, APP was first degraded to produce phosphoric acid, polyphosphoric acid and some other substances, which catalyzed the degradation and carbonization of EP composites, resulting in early decomposition of the EP composite. Meanwhile, INTs-PF_6_-ILs could facilitate this decomposition process, in order to obtain more char residue at high temperature. Moreover, the char residue could protect the EP composite matrix from further degradation and combustion, thereby leading to an incomplete degradation of the composite and improving the char residue rate of the composite at high temperature. Therefore, INTs-PF_6_-ILs showed a role of assisting the formation of char residue and protecting the EP composite from degradation.

### 3.3. CCT

**Figure 2:**
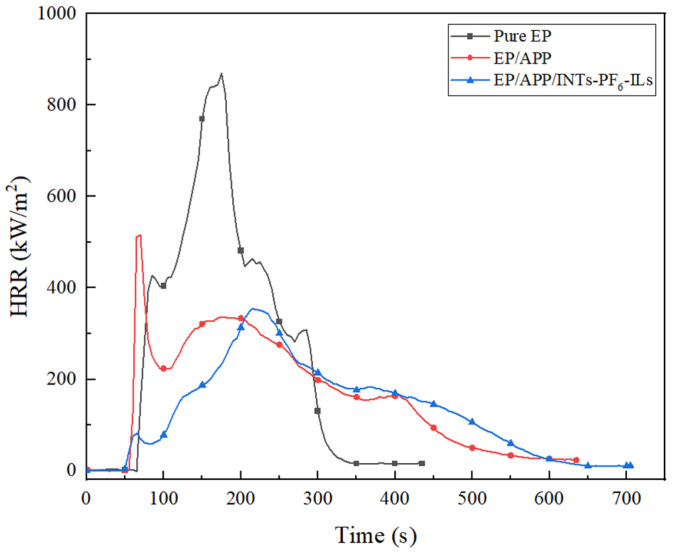
HRR curves of EP composites.

**Figure 3:**
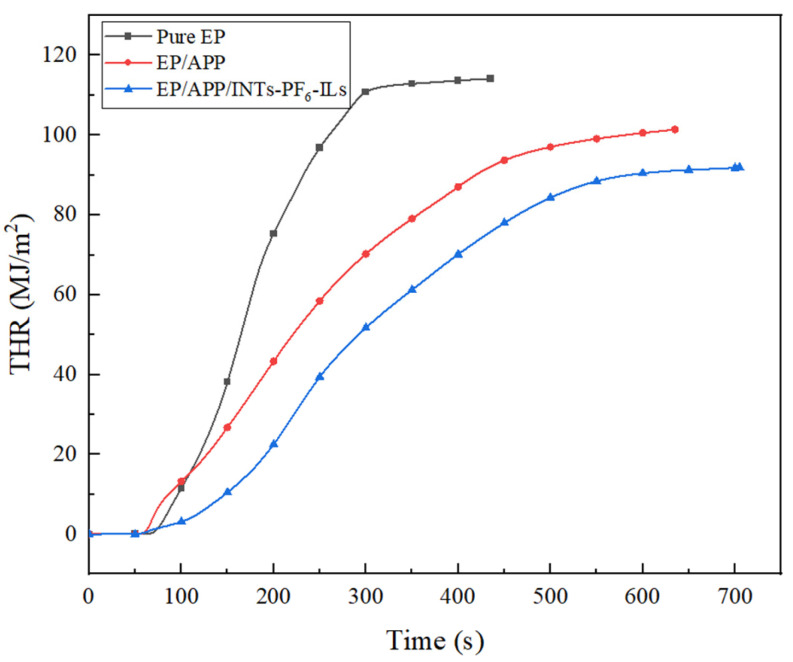
THR curves of EP composites.

**Figure 4:**
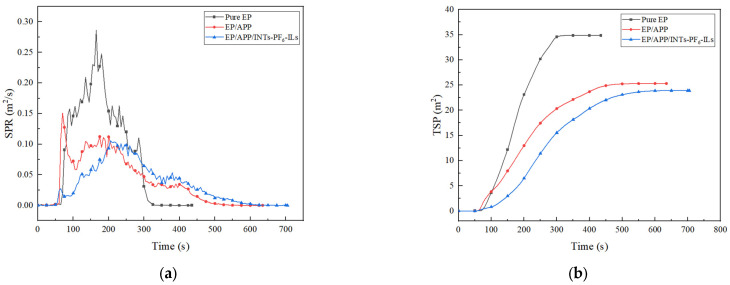
(**a**) SPR curves of EP composites; (**b**) TSP curves of EP composites.

**Figure 5:**
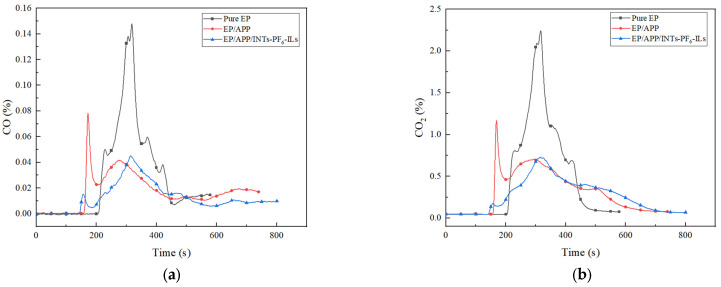
(**a**) Y_CO_ curves of EP composites; (**b**) Y_CO_2__ curves of EP composites.

Cone calorimeter was one of the most important and widely used instruments in the research of flame retardant polymer materials [29]. The related data obtained from CCT were shown in Figure 2, Figure 3, Figure 4 and Figure 5 and Table 4.

As described in Figure 2 and Table 4, the peak of heat release rate (PHRR) of flame retardant EP composites was reduced compared to the pure EP. The PHRR of EP/APP/INTs composites was decreased to 515 kW∙m^−2^ compared to the pure EP (869 kW∙m^−2^) by 40.74%. Moreover, the PHRR of EP/APP composites and EP/APP/INTs composites was very close to each other. The time to PHRR (T_PHRR_) of pure EP, EP/APP composites, and EP/APP/INTs-PF_6_-ILs composites was 175, 170, and 215 s, respectively. Apparently, T_PHRR_ of EP/APP/INTs-PF_6_-ILs composites was the longest among all the EP composites and prolonged by 40 s compared with the pure EP. The improved flame retardancy of EP/APP/INTs-PF_6_-ILs composites was due to the excellent synergistic flame retardancy of APP and INTs-PF_6_-ILs in EP composites, in which an excellent char layer could be formed with the catalytic carbonization of INTs-PF_6_-ILs, resulting in the efficient protection of the inside of the matrix from the erosion of oxygen and heat during combustion.

The fire performance index (FPI, FPI = TTI/PHRR) was an intrinsic property of materials [30]. Moreover, the larger the FPI value (FPI > 1), the lower the fire risk. The fire spread index (FSI, FSI = PHRR/T_PHRR_) represented the development of fire spread [31]. In contrast to the FPI, the higher the FSI value, the greater the fire risk.

In comparison with the FPI value of pure EP (0.076), the EP/APP and EP/APP/INTs-PF_6_-ILs composites were increased to 0.114 and 0.101, respectively. Moreover, EP/APP/INTs-PF_6_-ILs composites were increased by 32.9% compared to the pure EP. In addition, compared with the FSI value of pure EP (4.966), the EP/APP composites were reduced by 38.9% to 3.035. Meanwhile, the FSI value of EP/APP/INTs-PF_6_-ILs composites was further reduced by 51.8% and 21.1% compared to the pure EP and EP/APP composites, respectively, which suggested that the EP/APP/INTs-PF_6_-ILs composites had the lowest fire risk and its flame retardancy had been dramatically improved. This result might be attributed to the excellent synergistic effect between APP and INTs-PF_6_-ILs, which was proven again.

As described in Figure 3 and Table 4, the total heat release (THR) values of pure EP composites rapidly increased to the maximum value (114 MJ∙m^−2^) after ignition. However, the THR value of EP/APP composites and EP/APP/INTs-PF_6_-ILs composites was decreased to 101 and 92 MJ∙m^−2^, respectively. Compared with the EP/APP composites, the THR of the EP/APP/INTs-PF_6_-ILs composites was decreased by 9%, which evidenced that INTs-PF_6_-ILs could inhibit the heat release, and then enhance the flame retardancy of the EP composites.

The smoke production rate (SPR) and the total smoke production (TSP) of EP composites were shown in Figure 4. The peak SPR values of pure EP, EP/APP composites, and EP/APP/INTs-PF_6_-ILs composites were 0.286, 0.112, and 0.106 m^2^/s, respectively. In addition, the peak SPR values of EP/APP composites and EP/APP/INTs-PF_6_-ILs composites were reduced by 61% and 63%, compared with pure EP, respectively. Moreover, the TSP values of pure EP, EP/APP composites, and EP/APP/INTs-PF_6_-ILs composites were 37.4, 25.2, and 23.9 m^2^, respectively. Furthermore, the TSP values of EP/APP composites and EP/APP/INTs-PF_6_-ILs composites were reduced by 33% and 36% compared with pure EP, respectively. Considering that a large number of deaths in fires were caused by suffocation due to smoke inhalation, the reduction in smoke was crucial. Meanwhile, both of the values of peak SPR and TSP of EP/APP/INTs-PF_6_-ILs composites were the lowest among the EP composites. The possible reason may be attributed to the fact that the better char layer may inhibit the escape of smoke particles, which was generated by the decomposition of the EP composites.

The CO yield (Y_CO_) and CO_2_ yield (Y_CO_2__) were also important parameters for evaluating the fire safety of polymeric materials. In addition, the curves of Y_CO_ and Y_CO_2__ with time were shown in Figure 5. It was clear that Y_CO_ of pure EP quickly reached the peak, which was the highest of all samples, and the Y_CO_2__ of pure EP was the same. This was due to the fact that EP was flammable and could easily release a large amount of smoke including a large amount of incomplete combustion. In contrast, both the Y_CO_ and Y_CO_2__ of EP/APP composites were decreased significantly. This can be due to the obstruction of char layer formed by the APP promoting polymer decomposition and charing during burning. The rapidly formed char layer could effectively reduce the release of toxic gases. Meanwhile, it was decreased to a certain degree when INTs-PF_6_-ILs was incorporated, compared with the EP/APP composites. Therefore, EP/APP/INTs-PF_6_-ILs composites had the least toxic gas production, which was preferable. This result proved that the flame retardancy of EP could be improved with the synergistic effect of APP and INTs-PF_6_-ILs.

### 3.4. Morphology of Char Residues

**Figure 6:**
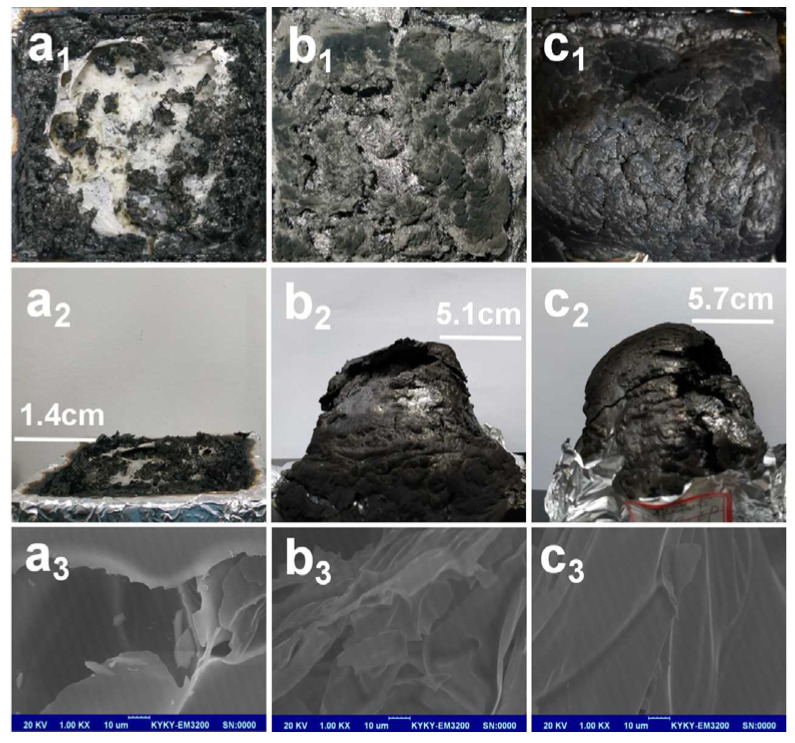
Digital photos and SEM images of residual char after CCTs. Herein, (**a_1_**,**b_1_**,**c_1_**) are the top view of digital photos of the pure EP, EP/APP, and EP/APP/INTs-PF_6_-ILs composites; (**a_2_**,**b_2_**,**c_2_**) are the side view of pure EP, EP/APP, and EP/APP/INTs-PF_6_-ILs composites; (**a_3_**,**b_3_**,**c_3_**) are SEM images of pure EP, EP/APP, and EP/APP/INTs-PF_6_-ILs composites.

Figure 6 presented the photographs of char residues after cone calorimeter tests. The top view of digital photos (Figure 6(a_1_,b_1_,c_1_)), side view of digital photos (Figure 6(a_2_,b_2_,c_2_)), and SEM images (Figure 6(a_3_,b_3_,c_3_)) at 1000 times magnification were used to evaluate the macro- and micro-morphology of the residual char surface of all the EP composites after the CCT.

As shown in Figure 6(a_1,_b_1_), there was a very small char layer for the pure EP, where the tin foil at the bottom could be observed, and the char residue height was only 1.4 cm. Meanwhile, the only char residue was covered with intensive holes, which was fragmented and brittle. In addition, even large holes and cracks could be observed from its micro-morphology (Figure 6(c_1_)). This residual char was very poor to resist the erosion of heat and combustible gas.

In the case of EP/APP, digital images of EP/APP composites showed a black, glossy, and expanded char residue, and the char residue height was up to 5.1 cm, which was increased by 264% compared with the pure EP. It demonstrated that the addition of 4 wt% APP could promote the EP to form a more integrated swollen residue char and the char residue of EP/APP composites was more compact compared with the pure EP, although some large holes were visible on the surface of the char residue. Furthermore, SEM images showed that the surface of char residue for EP/APP was continuous with few holes and cracks. Therefore, the char layer structure of EP/APP composites provided a better barrier for heat and flammable gas exchange, which may greatly affect the flame retardancy of the composites. However, it was fragile and loose.

After the introduction of INTs-PF_6_-ILs into EP/APP composites, the char residue height was up to 5.7 cm, which increased by 307% compared with the pure EP and by 12% compared with the EP/APP composites. Moreover, the char residue was more dense and solid and there were almost no holes or cracks on the surface. Furthermore, the surface was slightly rough, but continuous and highly complete. In this case, the better char residue structure and expansion ratio for EP/APP/INTs-PF_6_-ILs composites were the affirmed important reasons for its better flame retardancy. Therefore, not only the amount of the char residue was increased, but also the quality was strengthened. This char residue could protect the underlying materials from further burning and pyrolysis during the burning process, corresponding to the TGA and CCT.

## 4. Conclusions

In this work, INTs-PF_6_-ILs were introduced into the EP/APP system to increase its flame retardant properties. A good synergistic effect on the char formation and anti-dripping behavior of EP was found between INTs-PF_6_-ILs and APP, which results in a better flame retardant performance. The EP composites with 3.7 wt% APP and 0.3 wt% INTs-PF_6_-ILs could pass the UL-94 V-0 rating without dripping behavior. Moreover, the LOI value was 28%, which was higher than the EP/APP composites. The CCT results of EP/APP/INTs-PF_6_-ILs composites revealed that the PHRR (515 kW/m^2^) was the lowest, the THR (92 MJ/m^2^) was the smallest, and the TSP (23.9 m^2^) was the least. In addition, compared with the EP/APP composites, less toxic gas was released in the EP/APP/INTs-PF_6_-ILs. Moreover, the char layer formed by EP/APP/INTs-PF_6_-ILs was continuous and dense without holes, and it could resist the erosion of heat and gas and better protect the inside of the matrix. Therefore, the EP/APP/INTs-PF_6_-ILs composites had excellent flame retardant performance, which was attributed to the synergistic flame retardancy between INTs-PF_6_-ILs and APP in EP.

## Figures and Tables

**Table 1:** The composition of EP composites.

Sample	EP (wt%)	Curing Agent (wt%)	APP (wt%)	INTs-PF_6_-ILs (wt%)
Pure EP	88.9	11.1	-	-
EP/APP	85.3	10.7	4	-
EP/APP/INTs-PF_6_-ILs	85.3	10.7	3.7	0.3

**Table 2:** LOI and UL-94 rating of EP composites.

Samples	LOI (%)	UL-94
Pure EP	22.9	-
EP/APP	27.4	V-1
EP/APP/INTs-PF_6_-ILs	28.0	V-0

**Table 3:** Thermogravimetric analysis data of EP composites.

Samples	Air Atmosphere		
	T_i_ (°C)	T_max_ (°C)	Char Residue Rate (%) at 735 °C
Pure EP	355	359	15.06
EP/APP	336	342	18.83
EP/APP/INTs-PF_6_-ILs	337	343	19.61

**Table 4:** The characteristic data tested by the cone calorimeter.

Samples	Pure EP	EP/APP	EP/APP/INTs-PF_6_-ILs
TTI/s	66	59	52
PHRR/kW∙m^−2^	869	516	515
Time to PHRR/s	175	170	215
THR/MJ∙m^−2^	114	101	92
TSP/m^2^	34.7	25.2	23.9
FPI	0.076	0.114	0.101
FSI	4.966	3.035	2.395
EHC/MJ∙kg^−1^	21.3	21.7	21.3

## Data Availability

Not applicable.
